# How interprofessional simulation supports medical students’ transition to clinical practice

**DOI:** 10.1186/s41077-025-00392-w

**Published:** 2026-01-08

**Authors:** Libby Thomas, Gabriel Reedy

**Affiliations:** 1 Blizard Institute, Queen Mary University of London (QMUL), London, United Kingdom; 2 King’s College Hospital NHS Foundation Trust, London, United Kingdom; 3 Faculty of Life Sciences and Medicine, King’s College London, London, United Kingdom

**Keywords:** Interprofessional simulation, Realism, Confidence, Noticing, Threshold concept, Legitimate peripheral participation, Interdependent agentic capabilities, Undergraduate education, Multi-disciplinary, Behavioural skills, Health outcomes

## Abstract

**Introduction:**

Interprofessional education (IPE) improves outcomes for staff and patients, yet most pre-licensure training remains siloed. Simulation-based education (SBE) enhances clinical and communication skills, with particular benefit during transitions in training. While immersive simulation is costly, could integrating IPE and SBE at key transition points optimize its educational impact?

This study explores how interprofessional simulation can support transitions to practice, and what unique value it can contribute to the learning experience.

**Methods:**

This phenomenological study explored medical students’ experience of an interprofessional SBE programme. Adopting a sequential, mixed-methods design, students were asked about their experiences of participating in interprofessional simulations using various data collection methods over their transition from pre-licensure to professional practice. Methods included surveys (*n* = 229), one-on-one interviews (*n* = 29), focus groups (13 participants) and 1-year follow up interviews (*n* = 7). The qualitative data from interviews and focus groups are reported here. Codes and meaning units were developed and then scrutinized to develop a nuanced understanding of how the elements of the interprofessional simulation experience intertwined and the impact this had on the students.

**Results:**

Three overarching themes were identified from the analysis and were explored in greater detail: *realism*, *gaining confidence* and *learning to observe (noticing).* Interprofessional SBE provides the realistic environment for a unique educational experience in which students develop skills of observing interprofessional clinical practice, and can gain confidence for the transition into that practice. The implications are that the time and effort invested in overcoming the obstacles to offering interprofessional SBE lead to learning outcomes that may not otherwise be achieved. The results are further discussed through three theoretical lenses: Meyer and Land's threshold concepts, Richards' interdependent agentic capabilities and Lave and Wenger's legitimate peripheral participation.

**Conclusion:**

Interprofessional simulation offers students immersive, emotionally resonant experiences that enhance confidence, professional identity, and integration into clinical teams. These simulations prompt reflection on future roles and foster deeper understanding of interprofessional dynamics. The study identifies specific attributes of simulation that meaningfully contribute to learning, extending current SBE literature. By using a qualitative lens, this research highlights the unique value of interprofessional SBE in preparing students for collaborative practice and the complexities of modern healthcare.

**Supplementary Information:**

The online version contains supplementary material available at 10.1186/s41077-025-00392-w.

## Introduction

Healthcare is a challenging, complex and open system, in which well-trained professionals from different backgrounds work together to undertake increasingly complex patient care [1]. The need to work collaboratively in teams is widely recognised as beneficial to healthcare delivery and patient safety [2], and has led to the development of the field of interprofessional education (IPE).

Training professionals to work together in this complex healthcare landscape requires healthcare educators and professional regulators to generate appropriately contextualised IPE-related competencies and learning outcomes [3]. This has led to the emergence of a number of interprofessional competency frameworks [4–8] featuring common themes, including collaborative integrated care, role clarification, communication and reflection [3].

Systematic reviews have repeatedly shown that IPE helps team members to develop an appreciation their own and other team members roles more deeply, so that they better understand what each team member can contribute to patient care [9–11]. IPE leads to a change in collaborative behaviour and patient-centred healthcare, which together can result in improved patient safety. In addition, there is growing evidence that IPE contributes to Berwick’s [12] triple aims of healthcare reform: improved health of the population, improved patient satisfaction and reduced costs [13].

Although the case for IPE is compelling and the approach continues to grow in popularity, implementing IPE, especially in undergraduate and pre-licensure training, continues to be challenging [14–17], and there is less clarity about how to implement it with these learners [10, 18]. Pre-licensure training occurs primarily within the boundaries of each profession. This allows profession-specific training requirements to be met, and mostly avoids the logistical challenges IPE entails at a governmental, institutional and individual level [19]. As a result learners usually have limited opportunity to engage in formal IPE, especially in an experiential learning format [20]. This can cause problems when students qualify and start working as registered professionals: they may have little to no experience of interprofessional working to draw upon. With nursing, medical, and other health professions curricula necessarily full of content, there is little appetite or opportunity to “add IPE” as an additional piece within most health professions curricula.

If used appropriately, simulation is adept at enhancing team performance [21], preparing healthcare professionals to manage complexity [22] and improving healthcare systems through translational simulation [23], “whose purpose is to directly improve patient care and healthcare systems, through diagnosing safety and performance issues and delivering simulation-based interventions.” [24].

Simulation can also help specifically with the often stressful transitions between stages of training and practice. Empirical evidence underscores the utility of simulation to prepare pre-licensure students for the transition into professional practice [25–28] and for supporting qualified professionals as they transition along the career pathway and into new roles [29].

What happens when IPE and Simulation comes together? Sezgin and Bektas [14] showed that the combination of simulation and IPE led to improved teamwork and communication, but call for further studies to understand the long term impact of this potentially powerful combination of approaches. They also lay down the challenge that reported outcomes are not linked to theory, making it difficult to articulate why changes occur, or what conditions of the simulation may lead to positive outputs. Marion-Martins and Pinho [15] made a specific call for greater understanding and reporting of “how scenario activities contribute to the development of each competency factor… For instance, competencies, such as collaborative roles and conflict management, were investigated, but it was not clear how the scenario offered the opportunity to develop these competencies.” ([15], p.4).

Could we enhance the potential educational value of simulation by combining these concepts and constructs in particular moments of training, such as key transitions? If so, we may be able to provide much more value than is commensurate to the cost. Drawing together an interprofessional group of learners into SBE at the undergraduate level, despite the inherent challenges in doing so [30], provides an opportunity for learners to experience the rich complexity of interprofessional clinical practice in a scaffolded way that could help prepare them more effectively for the realities of clinical practice [15].

Research has often sought to describe or answer questions about the effectiveness of SBE [31] through evaluating either the student learning outcomes or evaluating the interprofessional initiatives themselves [20]. But questions remain about how interprofessional simulation can smooth and enhance the transition to practice. What is the unique educational value it offers, beyond experiences they get elsewhere in their training, that may make it worthwhile?

In this work, we sought to answer these questions by exploring medical students’ experiences of an IPE simulation programme that is purposefully situated at an important point of transition from pre-licensure to professional practice. In doing so, this research aims to understand more about the impact of simulated interprofessional participation on medical students' approach to their clinical placements and transition to qualified practice, and to understand what specific attributes of interprofessional simulation, if any, may potentially contribute to their learning. Finally, we respond to the frequent calls within the literature that empirical research should be grounded in, and should generalize back to, theory. In the discussion, we try to make sense of medical students’ experiences in simulation through different theoretical lenses, seeking further clarity, and better understanding of how, where and when, and in what situations simulation might be most valuable [32].

## Methods

### Methodological framework

This study was shaped by its overarching objectives and adoption of a phenomenological paradigm, which seeks to understand the nuanced dimensions of the students' experiences [33]. A sequential mixed methods design was adopted, in which the determination of emphasis in both data collection and analysis is imperative and should be carefully aligned with the research question and desired outcomes [34]. This manuscript reports on the qualitative data garnered from the interviews and focus groups, which were informed by the antecedent survey data, researcher-generated observations, and field notes.

This research adopts Walsh’s [35] comprehensive typology of reflexivity to inform a transparent and iterative research process [36]. The methodology aligns with hermeneutic phenomenology [37], underpinned by a constructionist epistemology [38] and an interpretivist paradigm [39]. The lead researcher, positioned as an educationalist within a healthcare context, engaged in continuous methodological and personal reflexivity throughout the project.

Although the SBE was interprofessional, this research specifically explored the lived experiences of the medical students, through their particular lens as members of the interprofessional team, and in the context of their training as undergraduate students. The time spent working in the clinical environment varies significantly between medical, nursing and midwifery students in the UK. While nursing and midwifery students are, by design, fully immersed in their respective roles early in their training, medical students spend less time in clinical practice and are typically in a more peripheral role to the team. Therefore, medical students’ experiences, perspectives and understanding of interprofessional working is likely to be very different from nursing or midwifery students.

### Context, setting, participants, and the IPE simulation

**Fig. 1:**
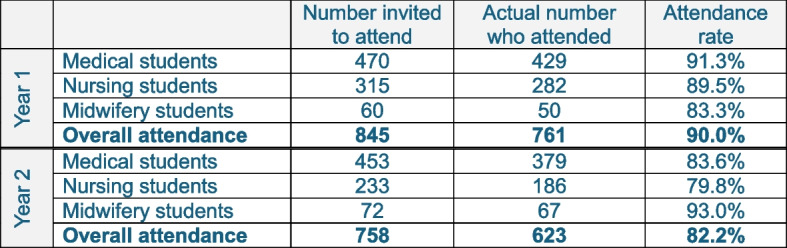
Total numbers of students engaging in the interprofessional final year simulations over the 2 consecutive years when survey data was collected

Final-year medical, nursing, and midwifery students at one of the largest health professions universities in Europe participated in a half-day simulation (Fig. 1). For the vast majority, this was their first experience of full patient simulation with advanced manikins. Sessions focused on preparing them to work as collaborative members of interprofessional care teams, and took place at purpose-built simulation suites across the university sites.

Ethics approval was granted by the Education and Management Research Ethics Panel at King’s College London.

Students were asked about their experiences participating in the interprofessional simulations using various methods: surveys, one-on-one interviews, and focus groups. Each method sought to explore particular aspects of the student experience. In line with data protection requirements, survey data was collected anonymously through an online survey tool. Audio recordings of the interviews and focus groups were transcribed and students’ names in the text were replaced with a unique code and identifying features were removed. Electronic data was stored in encrypted files.

The interprofessional SBE (detailed in Table 1, following reporting guidelines for healthcare simulation research as set out by Cheng et al. [40]), consisted of group introductions, an explanation of logistics and learning objectives, an overview of teamwork concepts, and three full-patient simulation scenarios. The three scenarios consisted of adult patients with: hypovolaemic shock from blood loss; seizure secondary to low blood sugar; and an anaphylaxis reaction to a wrong prescription.

**Table 1:** Key components of the interprofessional SBE (following from reporting guidelines for healthcare simulation research as set out by Cheng et al. (2016)) [40]

Participants:	Undergraduate nursing, midwifery, and medical students in groups of six to ten learners. Each group was split as evenly at possible between medical students and nursing or midwifery students. Nurses and midwives were on different courses but both with medical students to allow suitable patient scenarios to play out
Participant orientation:	The sessions started with introductions and an icebreaker followed by a welcome lecture introducing learning objectives, outlining the debrief format, and ending with a hands-on orientation to the environment including the manikin and the simulated ward. The aim was to help establish a psychologically safe learning environment
Simulator type and environment:	A Laerdal SimMan 2 or 3G manikin was placed in a simulated emergency department environment with relevant equipment including patient monitoring, drugs, fluids, patient notes and drug charts. A faculty member, via a microphone under the patient’s bed, provided the patient voice. An embedded simulation participant, in the role of a nurse, provided additional information and guidance at the bedside as needed. Additionally, the anaphylaxis scenario had an embedded simulated patient playing a role as a relative
Simulation event:	Half-day sessions comprised of three interprofessional simulated scenarios, each involving two to three participants. Other participants observed via video feed in the debriefing room. The intentionally broad learning objectives aimed to explore emerging behavioural skills. During debrief sessions, correct clinical management was elucidated, and facilitated discussions concentrated on identified behavioural skills, predominantly encompassing team working, communication, leadership, and followership
Instructional design:	The three scenarios were anaphylaxis, hypovolaemic shock and seizure. Each lasted approximately 10-min and was followed by a 15-min debrief. Each student participated in one interprofessional simulation scenario (medical student plus nurse or midwife student), unless non-attendance left space for a second opportunity. The students who took part in the anaphylaxis scenario subsequently completed a follow-on clinical communication scenario with the simulated patient acting as the distraught relative. The scenarios were adapted to make them clinically relevant to the midwives
Learning objectives:	To explore behavioural skills (sometimes called human factors) through student experiences in SBE. Any behavioural skills that arose as an interesting discussion point could be used but there was often a focus on: team working; communication; leadership; followership. The learning objectives were purposefully kept broad to allow a student-centred debrief to happen. The correct clinical management for each case was clarified before the facilitator steered the group to explore behavioural skills
Debriefing:	Facilitators were all trained to debrief using the Diamond [39] debrief model as the basic structure, with a paired co-debriefer if faculty numbers allowed. The interprofessional facilitators actively included all observers, as well as participants, in each debrief

Each scenario lasted approximately 10 min, followed by a 15-min structured debriefing using the Diamond model [41]. Non-participating members of the group actively observed the simulation via a video link, and then participated in the debriefing. Trained faculty members drawn from a mix of medical, nursing and midwifery educators led the debriefing, thereby maintaining sociological fidelity [42]. A debriefing aide memoire was available for facilitators, who had all completed at least a two-day simulation educator training course.

### Data collection and analysis

**Fig. 2:**
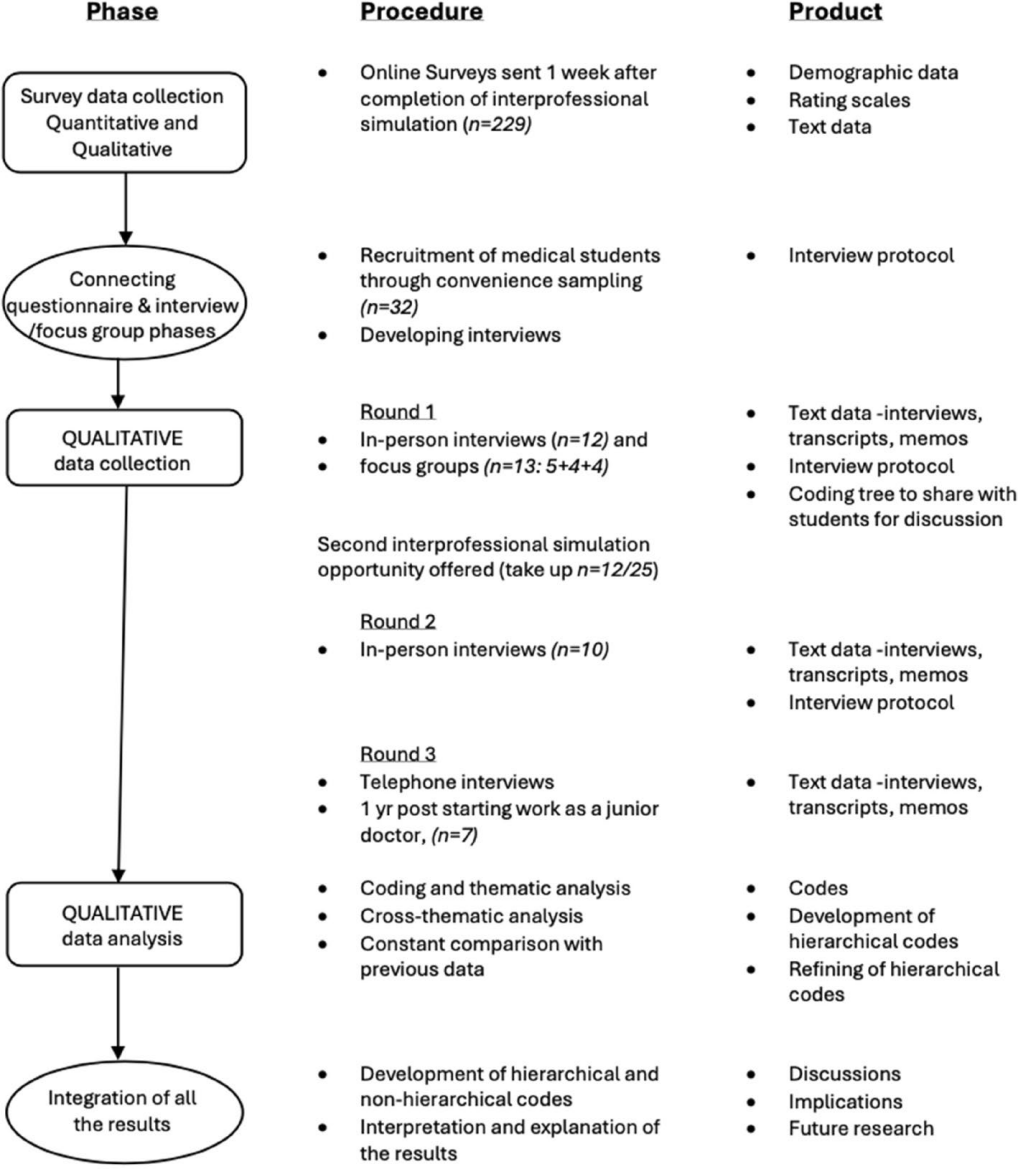
Visual model of the mixed-methods sequential design adapted from Ivankova et al. [43]

The research adopted a sequential, mixed-methods design [43], starting with an initial, comprehensive, overview through a survey (see Fig. 2 for study overview). The survey, which had been piloted and revised with a previous cohort of students, enabled the research to capture a broad spectrum of initial ideas from a significant proportion of the participating student cohort. A systematic coding process was applied, starting with survey data and eventually extending to the interview and focus group data reported here (Fig. 1). Acknowledging that the initial textual data was survey-based, the early coding process assumed both deductive and inductive forms [44]. This was the connecting point of the first and second stages of the study (Fig. 1) and also where integration of the data started to happen [34]. The survey data then informed the interview guide.

Subsequent stages involved a deeper exploration with a smaller cohort of participants through one-to-one interviews or focus groups, including follow-up interviews after the medical students had graduated and had begun their first year of professional practice. Recruitment criteria included willingness for post-simulation interviews or focus groups, with an additional SBE offered as an incentive. Individual interviews facilitate a collaborative exploration of the subject matter, enabling in-depth examination of areas of mutual interest between the interviewer and interviewee [45]. In contrast, focus groups facilitate in-depth discussions and diverse perspectives on the research topic through group dynamics, providing varied viewpoints and more critical commentary than individual interviews [46–48]. The use of focus groups in phenomenology can be seen as contentious but has become increasingly popular in healthcare research as a means of exploring new perspectives through stimulating group discussion [49].

The first and second interviews, conducted by the lead author, occurred within two to eight weeks after participation in the SBE, with the third interview conducted one year later. Using a semi-structured interview format (Additional File 1), interviews and focus groups were guided by topical interview schedules that were iteratively amended throughout subsequent rounds. This is in line with analytic induction, a system advocated by Strauss and Corbin [50] that involves a continuous process of collecting data in the field, conducting analysis on that data, then returning to the field to collect more data.

**Fig. 3:**
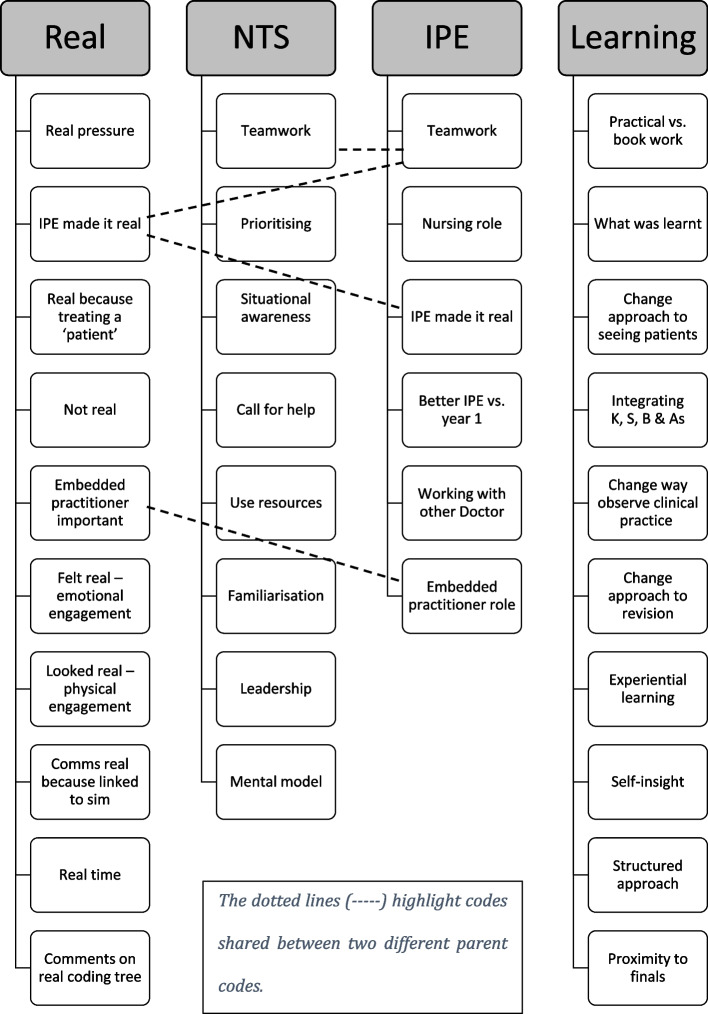
The hierarchical codes and their sub-codes developed from overall analysis of the data. The codes shown were given at the time which helped the researcher in the organization of the data

All data was read at least twice prior to coding allowing the researcher to engage in a dialogue with both text and participants [33]. MAXQDA software was used for systematic storage, organization, and analysis of qualitative data. Codes and memos were assigned to the data, utilizing a combination of non-hierarchical (flat) coding and hierarchical (tree) coding. In the latter, sub-codes facilitated the grouping of subsets under primary "parent" codes, as illustrated in Fig. 3. For instance, under the overarching code "Real," sub-codes such as "looked real – physical engagement," "IPE made it real," and "real pressure" were incorporated. The coding structure featured four original parent codes: "Real," Non-technical skills ("NTS"), Interprofessional education ("IPE"), and "Learning." Notably, certain codes directly pertained to two parent categories, indicated by dotted lines in Fig. 3.

Coding trees [51] shared emerging themes with interview participants, and facilitated discourse and the acquisition of diverse perspectives. This is a methodology in line with Gadamerian phenomenology that allows further dialogue with participants and the development of a deeper understanding of the phenomenon [33]. While all focus groups and initial interviews were conducted face-to-face, one-year follow-up interviews took place via telephone. All sessions were audio recorded, transcribed, and participants anonymized for analysis.

Acknowledging phenomenology’s philosophical roots and its absence of definitive analytical structure, this study integrated methodologies from other qualitative paradigms for a comprehensive data analysis approach. In the pursuit of qualitative rigor, both analytical sufficiency through rigorous analysis and data sufficiency in the richness in the data yielded was sought [47, 52]. A constant comparative method ensured revisitation and identification of previously overlooked yet significant statements, embodying a fluid and inductive approach to data generation and analysis [50, 53]. This continued until the data yielded no new significant insights, helping to create an argument for data sufficiency [50, 54].

During data collection and analysis, the researcher critically examined the influence of their professional identity—as a medical educator and clinician—on participants' responses. Given the existing power dynamics, including prior interactions through simulation training and the shared professional trajectory, it is acknowledged that students may have moderated their narratives. Strategies to mitigate this included conducting interviews in informal settings and inviting critical engagement with the coding framework to encourage openness.

Contextual reflexivity was also integral, particularly in light of evolving discourses in simulation and interprofessional education. The gap between data collection and publication prompted ongoing engagement with emerging literature to situate findings appropriately within current academic and clinical landscapes.

The codes and meaning units were further scrutinized to develop a nuanced understanding of how the elements intertwined so that the character of the phenomenon could be teased out. This determined that the essence of interprofessional simulation related to a combination of two of the meaning units: (a) The realism that the students’ described experiencing in the simulation and (b) the contribution that the interprofessionality of the simulation brings to the experience. These meaning units are explored in greater detail below through three overarching themes: *realism*, *learning to observe* and *gaining confidence*.

## Results

From the Year 1 cohort, 32 students were recruited for further participation, with 25 engaging in individual interviews (n = 12) or focus groups (n = 13). Ten participants took part in a second follow-up interview, and seven were available for a third interview post-graduation, following their transition into clinical practice.

Across all stages, students consistently reported that the realism of the simulation fostered deep engagement and facilitated the transfer of learning into clinical practice. This immersive experience was described as enhancing their confidence in patient care. Participants also highlighted the value of observing peers during interprofessional simulations and debriefs, which supported the development of reflective practice as they transitioned into clinical placements.

The core themes—realism, confidence-building, and observational learning—are explored in detail below, supported by illustrative excerpts from the data. Additional examples are presented at the end of the results in Tables 2, 3 and 4. All the quotes are taken from the qualitative data collected through interviews and focus groups.

**Table 2:** Additional qualitative data to support the theme 'Realism'

Realism	
Adil	“Well, as soon as you walk through the door, you have the feeling that something real [is] happening.”
Adil	“I mean, I never thought about this, but the environment actually makes a difference. If you're in the classroom, you're obviously in the classroom. And when you come in, and you've got a proper bed, a nurse, and then another doctor, or someone else, the phone, everything in one spot, and it looks like any other room in the hospital, it makes it really real.”
Charlie	“It’s more real because you can see, you know, you can look at the monitors, see things changing, you can actually put oxygen on, you can give drugs, you can see how those things change. I think that’s very useful.”
Jan	“I’ve never experienced anything like that before, so it’s…(like) a real person, with monitors going off and real time, so it was quite scary. …but it was very useful, the first time I’ve dealt with something like that wasn’t on a real person on a ward.”
Nori	“There is no, *“I would call 2222 *^a^*”.* You have to call quadruple whatever number it is. You can’t just keep saying, *“I would do this”.* You actually have to DO it. You have to wait for a response and you see the effects of what you are doing… So that is what makes it so realistic.”
Nori	“In a real life scenario you’re not always going to be surrounded by doctors. There might just be one nurse, or one healthcare professional, so that mixture, in that sense it was much more realistic. I wouldn’t have got so much out of the interprofessional communication if that hadn’t been the case, so it was very useful from that point of view.”
Jan	“…the real time, that does make a difference […] because you have to do something, not just say you’re going to do something, and it takes a lot more time than you’d expect.”
Darcy	“…you get more time than you think you have in that sort of emergency situation; you sort of think oh, I need to do this, bang, bang, bang, but it wasn’t… it was more sort of, well, we do this, and then would sort of see what happens next sort of thing.”
Luca	“I remember walking in and it was, it felt like a proper ward, and also the dummy was, if it was deteriorating, you know, we were all looking at each other, there was no-one else there, and I think that felt real as well.”
Kim	“You’re in this environment and there’s a lot going on. This person is deteriorating, and it does, you know, simulate it quite well. You do need to act relatively fast, to save this patient, and you’re not the only one with the pressure. There’s someone else who also feels the pressure, because they’re doing it with you. So, it does push you to try and think, what’s going on? How can we resuscitate this patient?”
Jas	“It was… really pressurised in a sense, you know, it imitated … real clinical commitments and… the kinds of things that you would see and experience and be challenged with.”
Mo	“I became aware very quickly of skills that I had but others did not and vice versa. I also became aware of how much authority I seemingly had as a doctor, which as a medical student I don't really have. I know now that in a real situation, tasks need to be shared and also communicated well.”
Garin	“(We were) given entire independence within the framework of the scenario, decisions we made would have real consequences that could have significant negative outcomes. Having this level of responsibility is something entirely new to us.”

**Table 3:** Additional qualitative data to support the theme 'Gaining confidence'

Gaining confidence	
Mary	“Gave me the confidence to discover that anything can be resolved with a structured approach and teamwork, even if it’s not immediately obvious what’s going on.”
Nori	“It highlighted to me who does what role. I was just always under the impression that the doctor did everything: took care of the obs, put in the cannulas, gave the oxygen. I hadn’t really thought about where the nurse fitted into the role or the healthcare professional I’ve had hardly any interaction with other healthcare professionals. Without that interaction you’re not aware of what their roles are. These learning situations, this is the first time I’ve had it.”
James	“It also gave me more confidence, as I felt that I was part of a team rather than trying to manage the situation alone.”
Isra	“I think, having done the simulations, you feel a lot more confident actually doing something, and I’ve been to two or three peri-arrests in the acute medical ward, and one of them was really serious, and there weren’t very many people around. As a result, you did feel like you can help rather than stand there and watch everyone else, try and do something.”
Rain	“I do feel a lot more confident now just going through the motions. Literally the thoughts are kind of translated into actions, which is really helpful We had a really sick patient who [had had a] horrible operation which went wrong, she just started to really deteriorate, and it was just me and my F1 and F2^a^. And we just kind of did it, and I knew then how to slot in to what they were doing. Because I knew what they were doing, because I had done it.”
Tyler	“It has certainly increased my confidence to get involved in potentially emergency situations, and has altered my view of my own role in such situations.”
Zuri	“I think you change your approach. Before you'd be, like, okay, this patient is bad; I'll go and get someone. Whereas now you'll look through things a little bit before you do that, so you take a small amount of responsibility for looking at obs and things like that and, giving initial small management decisions, making small management decisions, and then going to get someone.”
Jo	“My F1 was away looking at another patient on another ward, and the nurse comes and says to me “this patient’s not feeling very well”, and the other F1 wasn’t there. So I was the only person on the ward, so she was like, “can you come and have a look?” So I literally was like, okay, go back to ABCD; so I was by myself but then I did the whole initial assessment, and then afterwards then I gave her some oxygen and then when she was a bit stable I went to find my senior, and presented back; and then they did further investigations. So it was really useful to have [the simulation experience], and then all of sudden be thrown into it [a real-life scenario], so it was really useful, but, I think that [the simulation experience] really helped.”
Isra	“The multidisciplinary stuff is quite helpful because, having done it in the simulation, you know what other people are capable of doing and what they should be doing at different times”
Darryl	“The main thing I got was the communication between the different healthcare providers when you're in that situation.”
Nori	“I mean, it didn’t completely eradicate my nerves or anything, but it made me feel just that little bit more confident about starting in the first place, especially when attempting my first ever on-call. I remember thinking about the simulation quite a lot, actually, and just what I did and what I learned from it because I knew those same situations would probably be what I'd encounter on a ward
Frankie	“I just found it really useful doing the simulation because it gives you a point of reference if you came across an actual situation in real-life clinically. So it was quite nice to be able to refer back to the obvious fitting scenario and seeing where I went wrong and kind of trying to act a bit faster, recognise the situation a bit more quickly and what I need to do.”

**Table 4:** Additional qualitative data to support the theme 'Learning to observe'

Learning to observe	
Luca	“I was on an elderly care rotation, and when I went back to my ward, I saw how the nurses were working as well. So it was exactly [as] in the scenario – the nurses are usually the first people to pick up things, and then they forward it on to you.”
Nori	“I’m looking a lot more at how, especially the doctors, because that’s the position I’m going to be in, when they approach the patient, how they do that. They don’t just kind of go in straight to the patient, they always kind of go a little bit more slowly, take a look around. I have noticed that it’s very subtle, especially with experienced doctors. And again, their interaction with other healthcare professionals, not just nurses, but OTs, physios, healthcare assistants. They’re always gaining and giving knowledge to and from each other all the time. It’s a very dynamic process I hadn’t been so aware of it until I did the simulation.”
Jo	“…after you handle a situation you should maybe sit back and just think, if it didn’t go well, then you can think, why didn’t it go well? So, I guess, before I didn’t really think of like actually taking a step back and reflecting upon what went wrong, or maybe speak to the seniors and see what they would do differently; so, I think, it has been helpful in that respect, yes.”

### Realism

The shared lived experiences of the students illuminate the various manifestations of realism within the interprofessional simulation.

*“Well, as soon as you walk through the door, you have the feeling that something real [is] happening.” (Adil)*

The primarily semantic elements – those features of the simulation that become real only by mutual agreement of the participants through the establishment of a fiction contract [55] – were an important part of making the interprofessional simulation feel real. These included sounds from monitors signifying the patient was deteriorating, having to call for help on a telephone and wait for a response, and communicating within the interprofessional team.

*“I’ve never experienced anything like that before, so it’s…(like) a real person, with monitors going off and real time, so it was quite scary. …but it was very useful, the first time I’ve dealt with something like that wasn’t on a real person on a ward.” (Jan)*

Learning to work interprofessionally and learning about each other’s roles *vis a vis* their own roles, was a large part of the semantic element. Students described learning about the timeframes taken to react to changes and instigate treatments as they were given autonomy to work without senior support in the scenarios:

*“In a real life scenario, you’re not always going to be surrounded by doctors. There might just be one nurse, or one healthcare professional, so that mixture, in that sense it was much more realistic. I wouldn’t have got so much out of the interprofessional communication if that hadn’t been the case, so it was very useful from that point of view.” (Nori)*

The participants, including Nori, shared a pervasive feeling that the interprofessional simulation simulated the pressure of clinical practice and was anxiety inducing. They became emotionally involved to the extent that they felt the need to save the simulated patient’s life and perform to a high level so as not to let down the patient or team down.

Through their accounts, the students shared how the interprofessional simulation felt real, generated real emotions, and created a learning experience that had a profound effect upon them:

*“I became aware very quickly of skills that I had but others did not and vice versa. I also became aware of how much authority I seemingly had as a doctor, which as a medical student I don't really have. I know now that in a real situation, tasks need to be shared and also communicated well.” (Mo)*

Mo illustrates that simulation can offer clinical relevance without necessitating direct patient involvement. It establishes a fitting context for skill acquisition, immersing students in a clinical environment that authentically resonates with them, unveiling their professional and personal objectives, as well as challenges.

Because the patient is a simulated one, students can adopt a more central role in patient care: they can experience first-hand what it feels like to be responsible for a patient. That the patient is a manikin becomes inconsequential, as the students immerse themselves in the scenario and feel pressure to work alongside their interprofessional colleagues and care for the patient [56]. Interprofessional simulation can introduce ways to develop autonomy and to become independent, with self-directed learners maximizing on opportunities presented in simulation and on clinical placements to develop their agency.

*“(We were) given entire independence within the framework of the scenario, decisions we made would have real consequences that could have significant negative outcomes. Having this level of responsibility is something entirely new to us.” (Garin)*

### Gaining confidence

This study demonstrates a positive influence of the simulation on students' confidence as they navigate the transition into clinical practice. The interprofessional simulation instilled confidence in their clinical skills and provided a foundation for structured approaches to clinical cases. It helped students to understand and appreciate other professionals’ roles, and this translated into increased self-confidence:

*“(The simulations) gave me the confidence to discover that anything can be resolved with a structured approach and teamwork, even if it’s not immediately obvious what’s going on.” (Mary)*

The simulation helped students to become team players, to become more central in clinical activities. Descriptions were shared by the students of how they gained confidence and felt able to become more engaged in the care of patients on their clinical placements and they described taking responsibility for patients within an interprofessional team while awaiting medical support as Rain explains:

*“We had a really sick patient who [had had a] horrible operation which went wrong, she just started to really deteriorate, and it was just me and my F1 and F2. And we just kind of did it, and I knew then how to slot in to what they were doing. Because I knew what they were doing, because I had done it.” (Rain)*

The data shows that interprofessional simulation can enable student-initiated patient care, stepping up when there were no other doctors around to take the lead, thereby developing their own agency.

*“My F1 was away looking at another patient on another ward, and the nurse comes and says to me “this patient’s not feeling very well”, and the other F1 wasn’t there. So I was the only person on the ward, so she was like, “can you come and have a look?”*

*So I literally was like, okay, go back to ABCD; so I was by myself but then I did the whole initial assessment, and then afterwards then I gave her some oxygen and then when she was a bit stable I went to find my senior. So it was really useful to have [the simulation experience], and then all of sudden be thrown into it [a real-life scenario], so it was really useful, but, I think that [the simulation experience] really helped.” (Jo)*

Further, students were able to reflect back and articulate how the simulation had helped their transitions into professional practice as they found the interprofessional simulation was a good way to prepare for being a doctor in an interprofessional team. Those who had done the interprofessional simulation found that recalling their simulation experiences helped them to feel more confident responding to the call of their interprofessional colleagues and working with them for the better patient outcomes.

If simulation is integrated into the array of interventions aimed at mitigating the stress inherent in the transition period, as proposed in the literature [29], the potential exists for these advantages to extend to enhanced patient care. This is grounded in the assertion that students, having undergone cognitive and emotional preparation through simulation, are better equipped for clinical practice.

### Learning to observe

The lived experience within the interprofessional simulation offered participants novel avenues to broaden their understanding of clinical practice. The data underscores how interprofessional simulation engendered a sustained reflection on the dynamics of the interprofessional team and the ability to notice the unique contributions each member brings to patient care beyond the simulation setting. Students articulate how the simulation cultivates heightened observational skills and facilitates continuous learning about clinical practice, noticing what is happening in front of them.

*“I was on an elderly care rotation, and when I went back to my ward, I saw how the nurses were working as well. So it was exactly [as] in the scenario – the nurses are usually the first people to pick up things, and then they forward it on to you.”(Luca)*

Students revealed employing reflective practice methodologies, mirroring the debriefing model, upon returning to their clinical placements:

*“…after you handle a situation you should maybe sit back and just think, if it didn’t go well, then you can think, why didn’t it go well? So, I guess, before I didn’t really think of like actually taking a step back and reflecting upon what went wrong, or maybe speak to the seniors and see what they would do differently; so, I think, it has been helpful in that respect, yes.” (Jo)*

This finding suggests that by instructing students in active learning through observation, we have the potential to broaden their access to diverse, previously unexplored sources of knowledge, such as observing the other members of the interprofessional team. Moreover, this approach may enhance their sense of personal agency, fostering a greater inclination to assume responsibility for their actions and learning.

## Discussion

The lived experience of the participants in this study, as articulated in this research, shows that the rich and realistic interprofessional simulation provided participants with unique educational value and new ways to expand their understanding and the confidence to further improve their practice. Although simulation is well-tested and shown to be useful in various transition-to-practice contexts [29, 57], this research shows how the integration of the interprofessional element even before licensure or graduation could help to develop learners’ confidence and, in doing so, help them be more prepared for the transition to clinical practice.

In their own words, learners explained that while there were many aspects to the simulation that contributed to this sense of realism—a sense they confirmed once on their clinical rotation and exposed to more clinical practice experiences with living patients—the interprofessional component of the simulation, the sociological fidelity [58] was unique and especially powerful in contributing to this learning experience, offering experiences beyond their usual training. Even though IPE is often logistically difficult to make happen, this research reinforces how valuable it can be for learners [14].

Finally, although group observation is a component of many SBE interventions, the value of observation in the process has been explored more in relation to the in-course process and effects [59–61] rather than the longer term effects of learning to observe or notice in clinical practice. Intentional development of observation skills—which are useful throughout both clinical placements and other aspects of clinical training—can help learners take more active advantage of the wealth of potentially valuable but not explicitly educational situations they may find themselves in beyond the simulation. One subsequent product this programme has shown, for example, is that scaffolding the observation process helps learners to focus their attention. A greater awareness of, and interest in, the observer role could potentially improve our understanding of how to harness this learning opportunity, and access to opportunities for observation could be expanded in future training programme, increasing students’ exposure to certain clinical presentations and rare occurrences. Students can then draw from these experiences to reflect on other people’s practices during debrief sessions.

### Generalizing back to theory

While theory helps inform research, research must also help us understand theory more deeply. We argue that three conceptual frameworks can serve as analytical lenses, facilitating a nuanced interpretation of the complex and interrelated nature of our overarching themes [62] and responding to the call from SBE literature that empirical research should be grounded in, and generalized back to, theory [32]. These frameworks, namely threshold concepts [63–65], interdependent agentic capacities [66] and legitimate peripheral participation [67], offer theoretical scaffolding to delve into the intricacies of the data generated in the context of interprofessional simulation. They help us see the data in three different ways that will be discussed in more detail below. In synthesizing these conceptual frameworks into our discussion, we aspire not only to unravel the complexities inherent in the findings but also to provoke novel perspectives and insights that contribute to an enriched understanding of the phenomena observed within the interprofessional simulation context.

Meyer and Land's [63–65] conceptualization of threshold concepts provides a lens through which we can delve into focal junctures in learning, where profound understanding and transformation occur. This framework aids in dissecting the transformative moments revealed in the data, offering insights into the critical shifts experienced by participants during interprofessional simulation, which can smooth and enhance the transition to practice. This pedagogical approach affords students an experiential journey across pivotal thresholds that are particularly germane to the cultivation of their professional identities, offering a secure environment detached from direct patient interactions.

The findings underscore instances where students distinctly articulated various transformative "threshold" moments induced by their participation in the interprofessional simulation, helping us to understand more deeply how our learners think and feel about simulation as an educational experience. Notably, this simulation modality afforded students an immersive understanding of the experiential intricacies intrinsic to the role of a healthcare professional as they described the opportunity to *“be in the doctor’s shoes”* (Jac). Post-simulation, students not only reported a perceptible shift in their perspectives on clinical practice but also a heightened awareness of the intricacies associated with interprofessional collaboration, through the inherent sociological fidelity of the scenarios [58]. For some, the simulation imparted a palpable sense of being very real, as if they had actively participated in the acute provision of patient care, thereby contributing to a profound shift in their professional outlook.

An alternative lens for examining this research is offered through Richards, Sweets and Billett’s [66] model of interdependent agentic capacities, which becomes instrumental in interpreting the interconnected nature of capabilities and actions within the interprofessional simulation context. *Agentic* derives from *agency*, which is one’s capacity to act autonomously, make personal choices and effect the environment. *Capacities* relates to one’s existing knowledge. This framework assists in unpacking how participants' agentic capacities collaboratively contribute to the observed outcomes, fostering a nuanced understanding of the interplay between individual agency and collaborative capacities, a benefit that is not routinely measured or sought in the simulation-based learning research.

**Fig. 4:**
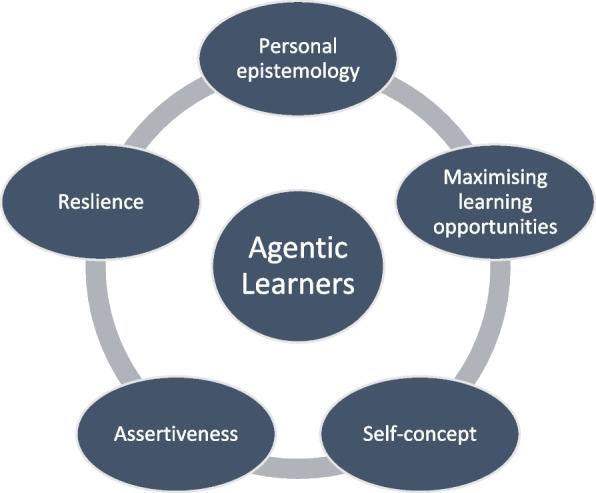
Interdependent agentic capacities as described by Richards et al. (2013) [66]

This conceptual framework delineates five interdependent factors integral to effectively engaging students in clinical education and fostering their agency, each of which is discernible in this study: assertiveness, maximizing learning opportunities, personal epistemology, resilience, and self-concept (Fig. 4).

Participants articulated how interprofessional simulation equipped them with the requisite tools for enhanced observation during clinical placements and facilitated reflective practices concerning both their own and others' clinical methodologies. These acquired skills were derived from techniques demonstrated in the debrief sessions and the observation of peers during interprofessional simulation scenarios. The students, throughout their participation, demonstrated a discernible evolution in their personal epistemology, leveraging and expanding their existing knowledge and ways of knowing. They exhibited a concurrent development of skills geared towards optimizing learning opportunities, achieved by actively immersing themselves in patient care activities and engaging in reflective practices that encompassed both observation and active participation.

Moreover, the heightened confidence reported by the students played a pivotal role in fostering a positive self-perception. This newfound self-assurance empowered them to assume a more active role in the clinical care of patients, characterized by assertive communication. The interprofessional nature of the simulation emerged as a catalyst, affording students valuable opportunities for reciprocal learning about their respective interprofessional roles and strategies for effective collaboration. In this context, peer collaboration within the interprofessional framework not only facilitated knowledge exchange but also laid the foundation for building resilience among students.

Finally, Lave and Wenger's [67] theory of Legitimate Peripheral Participation serves as a theoretical framework to elucidate the transformative process by which the students' enhanced observational or noticing skills and heightened confidence in participating in patient care contribute to their emerging professional identities as physicians. Lave and Wenger [67] posit that apprentices typically spend a considerable portion of their learning trajectory in a peripheral observational capacity, gradually assuming tasks of increasing significance and contribution. In the context of Legitimate Peripheral Participation, observation is deemed integral to comprehending the intricacies of a profession, providing a broader perspective on the objectives pursued by more experienced practitioners.

The students in this study frequently found themselves in a peripheral, observational role, initially undervaluing the learning potential inherent in this position. However, on reflection after the SBE, they gained an enlightened appreciation for the significant learning opportunities afforded through observation, particularly in understanding the role of a doctor and the nuances of effective team collaboration.

Furthermore, the students underwent an evolutionary journey, cultivating newfound confidence in their clinical capabilities. Confidence emerges as a pivotal component in determining students' readiness to engage in clinical scenarios and subsequently influences their performance therein. The augmented confidence resulting from the simulation empowers them to actively participate in clinical situations, transitioning from a peripheral to a more central role, thereby contributing substantively to patient care. This should encourage educators to believe that the trouble and expense of simulation-based learning can generate something genuinely meaningful to learners, leading to improved patient safety.

The power and importance of learning through observation is not a new concept to SBE [68], and numerous studies have found that observers in SBE can have a valuable learning experience, though often lesser than those who actively participate [61, 69, 70]. After observing a simulation, debriefing actively forces participants to acknowledge what they observed, consider, reflect and build upon these observations. The need to observe, and learn through observation, is a vital skill for clinical practice with simulation acknowledged to provide planned, structured opportunities to actively observe and thereby vicariously develop these skills [71].

Rooney and Boud [71] describe a “A pedagogy of professional noticing”: noticing in context, noticing of significance and noticing learning. And while “noticing can be seen as a component of reflection ….reflection literature tells us little about the noticing or observing phases of learning cycles” ([71] p. 445). The SBE literature to date does not currently explore if and how this ‘noticing’ extrapolates beyond the simulation learning experience, but this study has started to tease this out and articulate it. We highlight learning that is beyond experiences students have elsewhere in their training, specifically catalyzed through the interprofessional and immersive nature of the simulation.

From the data generated in this study, we do not claim that simulation in isolation induces transformative changes in students. We argue, however, that interprofessional simulation potentially constitutes a crucial component within a multifaceted array of educational opportunities and influences within their medical school training. It responds to Mario-Martins and Pinho [15] call for greater understanding of the ways in which SBE fosters development of collaborative competencies. Collectively, these influences contribute to the cultivation of students' confidence, fostering increased engagement during their clinical placements. This heightened engagement, in turn, extends their exposure to patients and facilitates the progressive development of their knowledge and skills.

By functioning as an integral element within this educational framework, interprofessional simulation serves to empower students, positioning them as more central participants within their prospective community of practice—the clinical team. The amalgamation of varied educational influences, of which interprofessional simulation is a vital part, collectively acts as an educational arsenal, strategically working towards the holistic development of students and their preparedness for active participation in the clinical domain.

### Strengths and limitations

This research was part of a larger doctoral study, which allowed an in-depth immersion in the data and fostered an iterative data analysis approach where a researcher (the primary author) conducted all the data generation and analysis. As such, it inevitably reflects the perspective of that researcher—who is, among other identities, a white, native-born woman working as an emergency medicine doctor at a major trauma centre in the UK, and who did this research while pursuing a doctorate in medical education. The offer of an additional SBE as an incentive for participation will have lead to selection bias of those who enjoy this style of learning, but this did also attract students willing to engage over a prolonged period of time leading to deeper understanding of the ongoing effects of the SBE.

The survey and interview questions were not developed as psychometric instruments, with constructs of reliability and validity as guiding principles, but being part of the interviews and focus groups seemed to prompt participants to reflect beyond what might be thought of as typical student engagement, which in turn could have enhanced their awareness and application of interprofessional simulation concepts. The presented results reflect all the expected features of context—that of the specific large urban university, simulation centre, and medical school at which this programme exists—thus potentially limiting the generalizability of the findings. Instead, the intention is to offer these findings as insights for researchers and educators, hopefully informing future research and improving interprofessional simulation experiences in other contexts.

## Conclusion

This study describes students' substantial and immersive encounters within interprofessional simulation, instilled with a palpable sense of realism. These experiences elicit the emotional responses anticipated in and integral to clinical practice, prompting contemplation of their future roles and those of their colleagues. On returning to clinical placements, students express newfound confidence, facilitating active engagement in patient care and improved integration into the interprofessional team. Additionally, students articulate the evolution of their clinical practice and an enhanced understanding of their future clinical milieu through novel observational and reflective approaches.

These findings helps us to understand the unique education value of interprofessional simulation, what specific attributes contribute to students learning and constitute significant contributions to the existing body of SBE literature, and builds upon established themes in prior interprofessional simulation studies across diverse contexts and participant groups. Leveraging a qualitative approach affords a comprehensive understanding of the nuanced factors influencing students, thereby enriching this critical domain of medical education. There is an imperative to prepare healthcare students for interprofessional collaboration and practice readiness to face the ongoing challenges of delivering safe and effective healthcare. Notably, this research emphasizes the indispensable and distinctive role of interprofessional simulation in achieving these preparatory objectives.

## Supplementary Information

Supplementary Material 1. 1^st^ Round Interview and Focus Group Schedule

## Data Availability

The datasets used and/or analysed during the current study are available from the corresponding author on reasonable request.

## Abbreviations

IPE: Interprofessional education
NTS: Non-technical skills
SBE: Simulation-based education
