# Supplementary material for: How interprofessional simulation supports medical students’ transition to clinical practice
Source: Adv Simul (Lond). 2026 Jan 8;11:2. doi: 10.1186/s41077-025-00392-w (PMC12784531; doi:10.1186/s41077-025-00392-w)
Supplement: Supplementary file 1 — Supplementary Material 1. 1st Round Interview and Focus Group Schedule [file 41077_2025_392_MOESM1_ESM.docx]

Additional File 1:

1^st^ round interview / focus group schedule

Interprofessional Clinical Simulation

Libby Thomas PhD

**Introduction**

My name is Libby Thomas and I am currently conducting some research into simulation training and the learning that goes on in and as a result of simulation.

I wanted to spend a bit of time with you today asking a few questions and exploring your thoughts on the simulation session you recently took part in – would that be ok?

I can assure that this conversation will be kept confidential.

I would like to record the session so that it can be transcribed at a later date, is that ok? The transcription will be anonymised.

**Q1.** Can I please ask you to confirm a couple of things with me before we start?

You are currently a final year medical student?

How old are you if you don’t mind me asking?

How long ago roughly did you participate in the simulation course?

And was this the first course of this type you had attended?

Was your course shared with nurses or midwives?

*(Exploring questions to use with following questions.*

*What about that interested you?*

*Would you be able to tell me a bit more about that?*

*What does this mean to you having thought about it?)*

**Q2.** What’s the first thing that comes to your mind when you think about your simulation experience?

And what was your overall impression of the simulation course?

As I mentioned, I’m studying how people learn during simulation. I want to explore a bit about your learning now.”

**Q3.** What do you think you learned on the course?

How did you learn that?

Why do you think that?

Can you give me an example?

(Exploring questions to use with following questions.

What about that interested you?

Would you be able to tell me a bit more about that?

What does this mean to you having thought about it?)

**Q4.** Can you think of an experience where you have put into practice what you learned on the simulation course?

Can you please describe it to me?

If no….

The simulation course comprised of several elements including the scenarios, the debrief, the communication session and the chance to work in a multi-disciplinary team.

Is there anything from any of those elements described that you have used from since you participated on the simulation course?

**Q5.** People have very different reactions to the simulation experience. Was there anything in the simulation experience that was negative for you?

**Q6.** The course includes a mix of trainee nurses/midwives and doctors.

How do you think that may have impacted on the learning experience for you?

**Q7.** Having experienced this course run with colleagues from nursing / midwifery, would you have preferred the experience to have been just medical students?

If yes,

What do you feel the advantages of that would have been?

Can you see if there would be any disadvantages?

If no,

Why not?

What was so important about the inter-professional aspect?

(Exploring questions to use with following questions.

What about that interested you?

Would you be able to tell me a bit more about that?

What does this mean to you having thought about it?)

**Q8.** Has the course altered your views on other members of the inter-professional team?

What I’d like you to do for just a minute is to project forward to when you start working on the wards as a junior doctor.

**Q9.** Has this experience made you think about where you will fit into the healthcare team?

The course was designed so that it encourages you to think carefully about how you performed in each situation.

**Q10.** What are your thoughts on whether this way of learning can help you with that or not?

Do you do think back on your performances differently now?

–in real practice as well as simulation.

In real life we go and talk about different experiences with our friends / colleagues over a coffee / in the pub etc

**Q11.** What kinds of things do you say, or do you hear your friends say, about the simulation course?

That is the end of the questions I have for you but is there anything else you would like to add?

Thank you for your time today, I really appreciate it. I may want to contact you again to clarify some of the comments you have made, would that be ok?

Added / extra questions

Would you take anything about the structure from the de-briefing back to clinical practice?

What do you think you took away from this learning experience that you have not gained anywhere else in medical school?

What can sim add to your curriculum that is not currently addressed?
